# Estetrol Inhibits Endometriosis Development in an In Vivo Murine Model

**DOI:** 10.3390/biom14050580

**Published:** 2024-05-15

**Authors:** Ana Sofia Zabala, Rocío Ayelem Conforti, María Belén Delsouc, Verónica Filippa, Maria Magdalena Montt-Guevara, Andrea Giannini, Tommaso Simoncini, Sandra Silvina Vallcaneras, Marilina Casais

**Affiliations:** 1 Laboratorio de Biología de la Reproducción (LABIR), Facultad de Química, Bioquímica y Farmacia, Universidad Nacional de San Luis, Instituto Multidisciplinario de Investigaciones Biológicas de San Luis (IMIBIO-SL-CONICET), San Luis D5700HHW, Argentina; asofia.zabala@gmail.com (A.S.Z.); ro.conforti64@gmail.com (R.A.C.); bdelsouc@gmail.com (M.B.D.); 2 Histología, Facultad de Química, Bioquímica y Farmacia, Universidad Nacional de San Luis, San Luis D5700HHW, Argentina; vpfilipp@gmail.com; 3 Consejo Nacional de Investigaciones Científicas y Técnicas (CONICET), San Luis D5700HHW, Argentina; 4 Molecular and Cellular Gynecological Endocrinology Laboratory (MCGEL), Department of Clinical and Experimental Medicine, University of Pisa, 56126 Pisa, Italy; magdalena.montt@gmail.com (M.M.M.-G.); andreagiannini06@gmail.com (A.G.); tommaso.simoncini@unipi.it (T.S.)

**Keywords:** endometriosis, estetrol, apoptosis, proliferation, hormone receptors, oxidative stress, mouse model

## Abstract

Endometriosis is characterized by the growth of endometrial-like tissue outside the uterus, and it is associated with alterations in the expression of hormone receptors and inflammation. Estetrol (E_4_) is a weak estrogen that recently has been approved for contraception. We evaluated the effect of E_4_ on the growth of endometriotic-like lesions and the expression of TNF-α, estrogen receptors (ERs), and progesterone receptors (PRs) in an in vivo murine model. Endometriosis was induced surgically in female C57BL/6 mice. E_4_ was delivered via Alzet pump (3 mg/kg/day) from the 15th postoperative day for 4 weeks. E_4_ significantly reduced the volume (*p* < 0.001) and weight (*p* < 0.05) of ectopic lesions. Histologically, E_4_ did not affect cell proliferation (PCNA immunohistochemistry) but it did increase cell apoptosis (TUNEL assay) (*p* < 0.05). Furthermore, it modulated oxidative stress (SOD, CAT, and GPX activity, *p* < 0.05) and increased lipid peroxidation (TBARS/MDA, *p* < 0.01). Molecular analysis showed mRNA (RT-qPCR) and protein (ELISA) expression of TNF-α decreased (*p* < 0.05) and mRNA expression of *Esr2* reduced (*p* < 0.05), in contrast with the increased expression of *Esr1* (*p* < 0.01) and *Pgr* (*p* < 0.05). The present study demonstrates for the first time that E_4_ limited the development and progression of endometriosis in vivo.

## 1. Introduction

Endometriosis is a chronic disease, characterized by the abnormal presence and growth of endometrial-like tissue outside the uterus, often accompanied by oxidative stress and inflammation. Endometriosis is estimated to affect 1 in 10 women worldwide of reproductive age, with 35–50% experiencing pelvic pain and/or infertility [1]. The origin of the disease is yet not fully understood due to its complex and multifactorial nature. The most accepted theory to explain the development of endometriotic lesions in a setting of oxidative stress and hypoxia within the peritoneal cavity is retrograde menstruation [2]. In this process, under the influence of several aberrant mechanisms, fragments of endometrial tissue implant, proliferate, and invade the peritoneum pelvic leading to the establishment of endometriotic lesions [3]. 

The involvement of endocrine dysregulation in the development of endometriosis is well established. Serum estrogen levels in patients with endometriosis do not vary compared to women without the pathology. However, endometriosis is considered estrogen-dependent due to increased local production of estradiol (E_2_) in the endometriotic tissue [4]. Additionally, estrogen receptor (ER) activity in endometriotic cells is altered. ERβ is often overexpressed, while ERα is down-regulated in endometriosis [5,6]. ERβ can represses ERα gene expression, and since E_2_ upregulates progesterone receptor (PR) expression through ERα, then the loss of ERα in endometriotic lesions can be followed up by a progesterone resistance state [7]. 

Although there is still no cure for endometriosis, patients with the disease can be treated surgically or pharmacologically. Treatment options for endometriosis include the use of non-steroidal anti-inflammatory drugs and hormonal therapies. Hormonal contraceptives are among the first-line therapies for managing the disease and its associated symptom due to their ability to reduce or suppress menstruation and/or ovulation. However, an increased risk of thromboembolic events and potential effects on breast tissue proliferation have been associated with the use of estrogens in combined oral contraceptives. Endometriosis is a chronic disease that requires long periods of treatment; therefore, the safety of an estrogen-based therapy is particularly important [8,9].

Estetrol (E_4_) is a weak natural estrogen produced exclusively by the human fetal liver [10]. E_4_ exhibits a 4-to-5-fold higher binding affinity for ERα than the ERβ, and 25-fold lower binding affinity for ERα compared to E_2_ [11]. Despite behaving as a weak agonist in bone, myometrium, and endometrium among other tissues, E_4_ is effective in reducing hot flush and inhibiting ovulation [12,13,14,15]. Notably, E_4_ displays agonist and antagonist activities on membrane ERα depending on the tissue. Based on this particular mode of action, E_4_ is considered to be a Native Estrogen with Specific Action in Tissues (NEST) [16,17].

Recently, Patiño-García et al. [18] demonstrated in human endometriotic cells lines and primary cultures from endometriotic patients that E_4_ increased the PR levels and progesterone gene response in endometriotic cells in culture, without affecting cell growth or migration. However, this study showed the necessity of incorporating a pathophysiological model where autocrine, paracrine, and endocrine regulations are present for a comprehensive in vivo analysis of the E_4_ role in endometriosis. 

Our aim was to evaluate the effect of E_4_ on the growth and development of endometriotic lesions in an induced endometriosis mouse model. Therefore, we examined proliferation and apoptosis of endometriotic cells, oxidative stress, TNF-α level, and hormone receptor expression.

## 2. Materials and Methods

### 2.1. Animal Handling

Female C57BL/6 mice, approximately two months old and weighing 19–21 g, were obtained from the animal facility at the Universidad Nacional de San Luis, Argentina. Mice were housed in environmentally controlled facilities at a controlled temperature range (22 °C ± 2 °C) and 12 h light–dark cycle [19,20,21]. All animal procedures were approved by the Comité Institucional de Cuidado y Uso de Animales of the Universidad Nacional de San Luis (Protocol No. B-384/21) in accordance with the Guide for the Care and Use of Laboratory Animals of the National Institutes of Health (8th ed., 2011, Washington, DC, USA).

### 2.2. Endometriosis Surgical Induction

The induction of endometriotic-like lesions was made through autologous transplant, following our protocols previously reported [19,20,21]. It should be noted that these animals do not menstruate and, therefore, do not develop the disease spontaneously. However, the murine model of endometriosis is widely validated by the literature for preclinical studies [22,23]. Briefly, animals were anesthetized with ketamine hydrochloride (100 mg/kg, Holliday Scott, Buenos Aires, Argentina) combined with xylazine chlorhydrate (10 mg/kg Richmond, Buenos Aires, Argentina) with an intraperitoneal injection. The right uterine horn was excised and cut into three small fragments of about 4 mm^2^. Tree uterine segments were attached to the mesentery with a single 6-0 black nylon knot.

### 2.3. Experimental Design and Sampling of Biological Materials

Sixteen female mice with induced endometriosis were randomly divided into two groups: the group that was treated with E_4_ and the control group that was treated with vehicle alone.

**Scheme 1:**
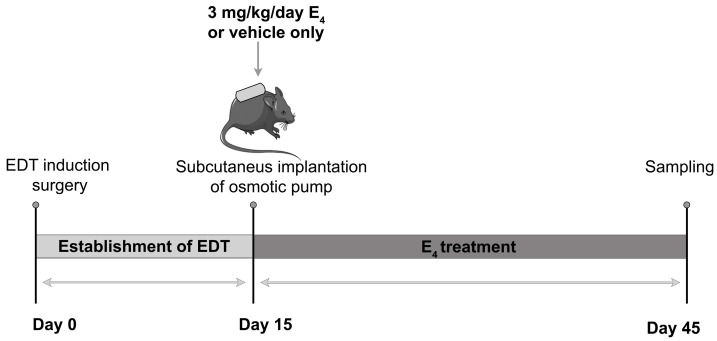
Experimental design. Surgical endometriotic-like lesion induction was made through an autologous transplant in female C57BL/6 mice. After 15 days of the establishment of endometriotic-like lesions, the treated group received subcutaneous mini osmotic pumps, releasing E_4_ at a dose of 3 mg/kg/day solved in the vehicle (polyethylene glycol + dimethyl sulfoxide). Control group received vehicle-only. Over 4 weeks of treatment, animals were euthanized and samples were taken. EDT: endometriosis, and E_4_: estetrol. The scheme was created in part using Servier Medical Art, provided by Servier, and licensed under a Creative Commons Attribution 4.0 unported license (https://creativecommons.org/licenses/by/4.0/).

At the 15th postoperative day, the time necessary for the establishment of endometriotic-like lesions [24,25], animals received subcutaneous mini osmotic pumps (Alzet, model 2004; 0.25 µL/h), releasing E_4_ at dose 3 mg/kg/day (Pantarhei Bioscience, Zeist, The Netherlands) solved in the vehicle (polyethylene glycol + dimethyl sulfoxide) for the following 4 weeks (Scheme 1). The half-life of E_4_ in rodents is 2–3 h. It has been reported the use of osmotic mini pumps in mice provides a constant level of circulating E_4_ for several weeks compared with subcutaneous, intraperitoneal, or oral administration of E_4_ [26]. The treatment duration and dose were based on previous studies that analyzed the effect of E_4_ alone [27,28] or in combination with E_2_ [29,30]. After 45 ± 2 days of experimental endometriosis induction, mice were euthanized, and samples were taken [19,20,21,31]. Mice were peritoneally injected with 1.5 mL of sterile phosphate-buffered saline (PBS). Peritoneal fluid was collected and centrifuged at 250× *g* for 10 min at 4 °C to remove cells and stored at −80 °C. Finally, endometriotic-type lesions were removed from intestinal mesentery for the subsequent analysis.

### 2.4. Macroscopic Study of Endometriotic-like Lesions

According to our previously reported protocols, endometriotic-like lesions were identified, counted, and measured for volume calculation [19,20,21]. Then, samples were removed and weighed. One lesion per animal was fixed in 4% paraformaldehyde for 24 h at 4 °C, embedded in paraffin, and sectioned at 4 μm thick for further histological analysis. The specimens were evaluated after routine hematoxylin–eosin staining to confirm the histological features of the endometriotic-like lesions (glands and stroma). The remaining lesions were stored in RNAhold^®^ (TransGen Biotech Co., Ltd., Beijing, China) to inactivate RNase and keep RNA intact for RT-qPCR analysis.

### 2.5. Proliferating Cell Nuclear Antigen (PCNA) Immunohistochemistry

PCNA is commonly used as a marker of cell proliferation in immunohistochemical studies due to its crucial role in DNA replication and repair [32,33]. PCNA immunohistochemistry was performed according to our previously reported protocols [19,20,21]. Briefly, histological sections from six different animals per experimental group were investigated. Sections were incubated with rabbit anti-mouse PCNA monoclonal antibody (1:1000; D3H8P, XP Rabbit mAb, Cell Signaling Technology, Danvers, MA, USA) in 1% BSA overnight at 4 °C. The sections were then treated with biotinylated goat anti-rabbit IgG antibody (1:900; B8895, Sigma-Aldrich, St Louis, MO, USA) followed by incubating with streptavidin-peroxidise conjugate (VectorLabs, Burlingame, CA, USA). The specific antigens were visualized with a diaminobenzidine (DAB) reaction (CellMarque, Sierra College Blvd Rocklin, CA, USA), counterstained with hematoxylin, and cover-slipped for microscopic examination. Negative controls were tested without the primary antibody. Proliferation was determined by counting the number of positively stained cells (brown nuclear reactivity) at 400× under standard light microscope. The mean percentage of proliferating cells was calculated from 4 to 6 representative fields per slide for each experimental group (*n* = 6 animals per group).

### 2.6. Terminal Deoxynucleotidyl Transferase-Mediated Nick End Labeling (TUNEL) Assay

Slides from endometriotic-like lesions were processed using the In Situ Cell Death POD kit (Roche, Mannheun, Germany) according to the manufacturer’s instructions. Sections were counterstained prior with hematoxylin and mounted. The percentage of TUNEL-positive cells (brown staining nuclei) was calculated by counting 4–6 representative fields from each slide for each experimental group (*n* = 6 animals per group) on a standard light microscope at 400× magnification. The mean percentage of death cells was calculated for each experimental group. A slide pre-treated with DNase I recombinant, grade I (Sigma-Aldrich, Saint Louis, MO, USA) was used as a positive control. Another histological section was incubated with label solution (without terminal deoxynucleotidyl transferase) as a negative control.

### 2.7. Antioxidant Enzyme Activities

Endometriotic-like lesions were homogenized in a medium containing 120 mM KCl, 30 mM phosphate buffer (pH 7.4), and 1% protease inhibitor cocktail (P8340, Sigma-Aldrich, St. Louis, MO, USA). Proteins were quantified according to the Bradford method using bovine serum albumin (BSA) as a standard (Sigma, St. Louis, MO, USA) [34]. Superoxide dismutase (SOD) activity was determined by measuring the capacity of the enzyme to inhibit pyrogallol autoxidation, monitoring the change in absorbance at 420 nm per minute. One unit of the enzyme was expressed as the amount of SOD that inhibited 50% of pyrogallol autoxidation [35]. Catalase (CAT) activity was assessed by monitoring the reduction in absorbance at 240 nm caused by hydrogen peroxide (H_2_O_2_) decomposition. One CAT unit equals the amount of enzyme needed to decompose 1 μM of H_2_O_2_/min [36]. The GPX activity was measured by monitoring NADPH oxidation at 340 nm [37]. The results were reported as units of enzyme activity per milligram of protein (U/mg protein). All measurements were performed in duplicate.

### 2.8. Measurement of TBARS-MDA

The thiobarbituric acid reactive substance (TBARS) method described by Draper and Hadley was used to determine the lipid peroxidation levels in homogenates of endometriotic-like lesions [38]. For malondialdehyde (MDA) standards, a stock of 1,1,3,3-tetraethoxypropane was used, and the absorbance was read under 530 nm wavelength. All measurements were conducted in duplicate, and the results were reported in μmol of MDA per milligram of protein (μmol MDA/mg protein).

### 2.9. Enzyme-Linked Immunosorbent Assay (ELISA)

Peritoneal fluid TNF-α concentrations were assessed in mice using a DuoSet ELISA Kit (R&D Systems, Minneapolis, MN, USA) according to the manufacturer’s instructions. The concentration of cytokines was calculated by linear regression.

### 2.10. RNA Extraction and Quantitative Reverse Transcription Polymerase Chain Reaction (RT-qPCR)

RNA isolation and RT-qPCR from endometriotic-like lesions were conducted as previously mentioned [20,21]. Briefly, total RNA was isolated using TRIzol reagent (Thermo Fisher Scientific, Waltham, MA, USA). RNA concentrations were quantified, purities were verified, and treated with RQ1 RNase-Free DNase (Promega, Fitchburg, WI, USA). Purified total RNA was reverse transcribed to cDNA using the Transcriptor First Strand cDNA Synthesis Kit (Roche Diagnostics International Ltd., Mannheim, Germany). qPCR amplification was performed with FastStart™ Universal SYBR^®^ Green Master (Roche Diagnostics International Ltd., Mannheim, Germany). Specific primers for *Esr1* (ERα), *Esr2* (ERβ), *Pgr* (PR), and *Tnf* (TNF-α) were custom-designed using IDT primer quest software (Table 1). All primer sets were tested and validated through melting curve analysis. A relative quantification method (2^−ΔΔCt^) was used to evaluate the expression of each gene corrected to 18S ribosomal RNA—*Rn18s*—as an endogenous control [39]. 

### 2.11. Statistical Analysis

The experimental data were processed using GraphPad Prism (Version 5, GraphPad Software LLC, San Diego, CA, USA). Data were expressed as mean ± standard error of the mean (SEM). All of the data were assessed using the Shapiro–Wilk test to determine if they were normally distributed. An unpaired two-tailed *t*-test was used to analyze the significance of differences between the two groups. For all comparisons, *p*-values < 0.05 were considered statistically significant.

## 3. Results

### 3.1. Effect of E_4_ on the Growth of Endometriotic-like Lesions

**Figure 1:**
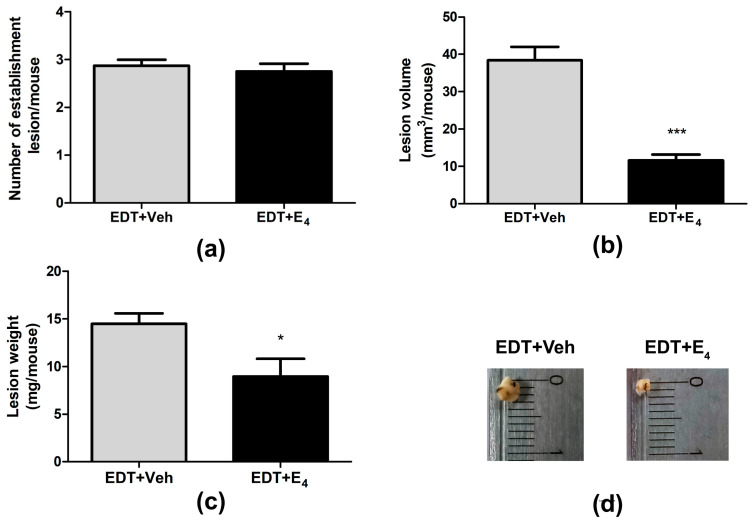
Effect of E_4_ on the growth of endometriotic-like lesions. In mice with induced endometriosis, the number of established lesions (**a**), their volume (**b**), and their weight (**c**) were examined after receiving either 3 mg/kg/day of E_4_ (EDT+E_4_) or vehicle-only (EDT+Veh). Mice were treated continuously for 4 weeks, from day 15 after endometriosis induction surgery to the day of euthanasia. Representative photography of endometriotic-like lesions treated with E_4_ or vehicle after endometriosis induction (**d**). Statistical significance was determined by Student’s *t*-test. All data are presented as mean ± SEM (*n* = 8 animals per group). * = *p* < 0.05 and *** = *p* < 0.001. EDT: endometriosis; E_4_: estetrol; and Veh: vehicle.

To investigate the effect of E_4_ on endometriosis, we first examined the establishment of the disease. Macroscopically, the number of established endometriotic-like lesions did not differ significantly between the experimental groups (Figure 1a); however, we found that E_4_ treatment reduced the volume and weight of the lesions compared to the control group (*p* < 0.001 and *p* < 0.05, respectively; Figure 1b,c), and this observation is supported by the representative photograph shown in Figure 1d. Our results suggest that E_4_ treatment restricts the progression of the disease in the experimental model. 

### 3.2. Effect of E_4_ on Cell Proliferating in Endometriotic-like Lesions

**Figure 2:**
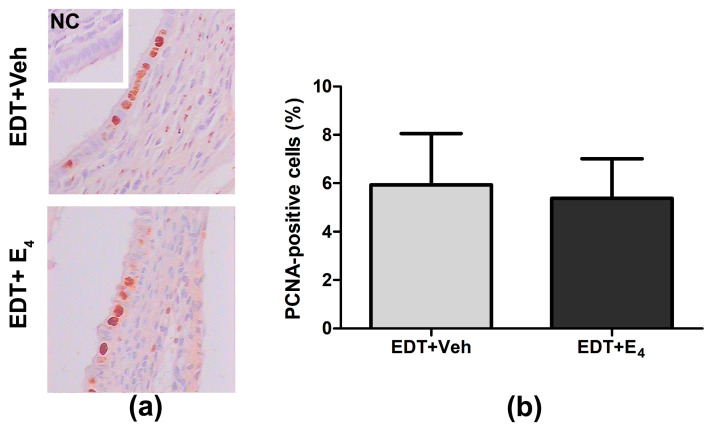
Effect of E_4_ on cell proliferating in endometriotic-like lesions. (**a**) Micrography of representative slices from immunohistochemical analysis of PCNA in endometriotic-like lesions from endometriosis-induced mice treated continuously for 4 weeks with E_4_ (EDT+E_4_) or vehicle-only (EDT+Veh). The inset shows PCNA-negative control (NC) incubated without primary antibody. Magnification: 400×. (**b**) Percentage of PCNA-positive cells (brown nuclear reactivity). Statistical significance was determined by Student’s *t*-test. All data are presented as mean ± SEM from 4 to 6 representative visual fields (*n* = 6 animals per group). EDT: endometriosis; E_4_: estetrol; and Veh: vehicle.

Endometriosis is a disease known to be E_2_-dependent, which promotes the cellular proliferation of endometriotic tissue [4]. Histologically, we confirmed the typical signal of endometriosis (gland and stroma). PCNA results showed that E_4_ did not have a statistically significant effect on cell proliferation of endometriotic-like lesions in both groups (Figure 2a,b). This result is promising, especially considering that the dose administered would not exacerbate the condition in our experimental model.

### 3.3. Effect of E_4_ on Cell Death in Endometriotic-like Lesions

**Figure 3:**
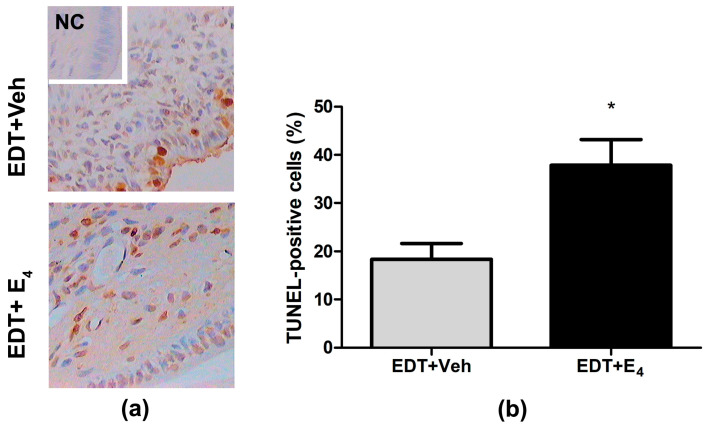
Effect of E_4_ on cell death in endometriotic-like lesions. (**a**) Micrograph of representative slices from the TUNEL technique in endometriotic-like lesions from endometriosis-induced mice treated continuously for 4 weeks with E_4_ (EDT+E_4_) or vehicle-only (EDT+Veh). The inset shows TUNEL-negative control (NC) incubated without terminal deoxynucleotidyl transferase. Magnification: 400×. (**b**) Percentage of TUNEL-positive cells (brown nuclear reactivity). Statistical significance was determined by Student’s *t*-test. All data are presented as mean ± SEM from 4 to 6 representative visual fields (*n* = 6 animals per group). * = *p* < 0.05. EDT: endometriosis; E_4_: estetrol; and Veh: vehicle.

The development of endometriosis has been shown to result in resistance of endometriotic cells to apoptosis [40,41]. The TUNEL assay was used to quantify dead cells. The results show a significant increase in apoptosis of lesions treated with E_4_ in endometriosis-induced mice compared to the control group (*p* < 0.05; Figure 3a,b). This finding provides evidence for the efficacy of E_4_ in treating endometriosis by promoting cell death and consequently reducing the volume and weight of endometriotic-like lesions. 

### 3.4. Effect of E_4_ on Oxidative Stress in Endometriotic-like Lesions

**Figure 4:**
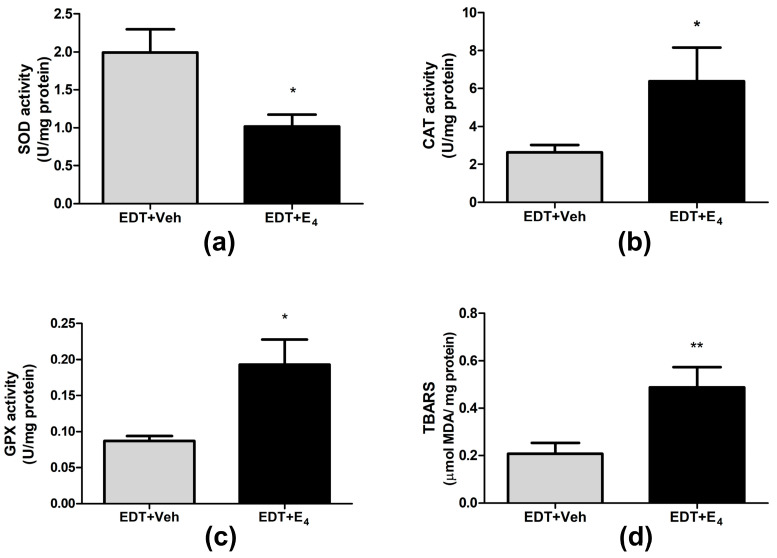
Effect of E_4_ on oxidative stress in endometriotic-like lesions. The activity of the antioxidant enzymes SOD (**a**), CAT (**b**), and GPX (**c**) and the levels of TBARS/MDA (**d**) were determined by spectrometry methods in endometriotic-like lesions from endometriosis-induced mice treated with E_4_ (EDT+E_4_) or vehicle-only (EDT+Veh) for 4 weeks. Statistical significance was determined by Student’s *t*-test. All data are presented as mean ± SEM (*n* = 8 per group). * = *p* < 0.05, and ** = *p* < 0.01. EDT: endometriosis; E_4_: estetrol; Veh: vehicle.

Since oxidative stress can modulate cell proliferation and apoptosis in endometriotic tissue [42], we explored the E_4_ effect on the key enzymatic antioxidants and the expression of an oxidative damage marker. According to the results obtained, the group treated with E_4_ decreased the activity of SOD (*p* < 0.05), while the activity of CAT (*p* < 0.05) and GPX (*p* < 0.05) increased, compared to the control group (Figure 4a–c). Furthermore, MDA concentration was higher in the E_4_-treated group (*p* < 0.01) compared to the control group (Figure 4d). These results suggest that E_4_ treatment disrupts redox status and promotes lipid peroxidation in endometriotic-like lesions, which supports the decrease in lesion volume and the increase in cell death evidenced in the TUNEL analysis.

### 3.5. Effect of E_4_ on TNF-α Expression in Endometriotic-like Lesions and Peritoneal Fluid

**Figure 5:**
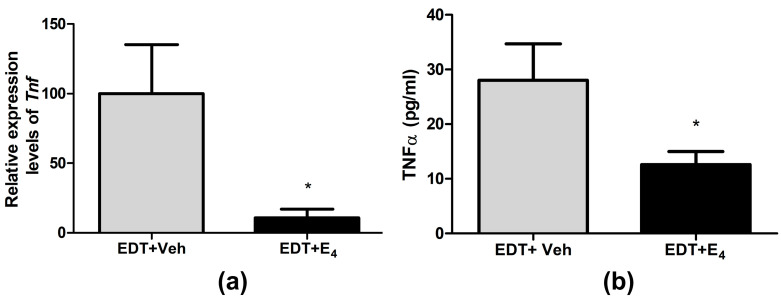
Effect of E_4_ on TNF-α expression. (**a**) Relative mRNA levels of *Tnf* were quantified by RT-qPCR in endometriotic-like lesions from endometriosis-induced mice treated for 4 weeks with E_4_ (EDT+E_4_) or vehicle-only (EDT+Veh). (**b**) TNF-α protein was quantified by ELISA in peritoneal fluid. Statistical significance was determined by Student’s *t*-test. All data are presented as mean ± SEM (*n* = 6 animals per group). * = *p* < 0.05. *Tnf*: tumor necrosis factor transcript variant 2; EDT: endometriosis; E_4_: estetrol; and Veh: vehicle.

As part of the study, we examined the effect of E_4_ on TNF-α expression. TNF-α is an inflammatory marker that contributes to the development and establishment of endometriotic implants [43]. It was observed that *Tnf* mRNA levels were significantly reduced in the endometriotic-like lesions of the group treated with E_4_ (*p* < 0.05; Figure 5a). In addition, the expression of TNF-α protein in the peritoneal fluid was also found to be reduced by E_4_ compared to the control group (*p* < 0.05; Figure 5b). These results suggest that E_4_ treatment has a down-regulatory effect on TNF-α in endometriotic-like lesions and peritoneal fluid.

### 3.6. Effect of E_4_ on Esr2, Esr1, and Pgr mRNA Expression in Endometriotic-like Lesions

**Figure 6:**
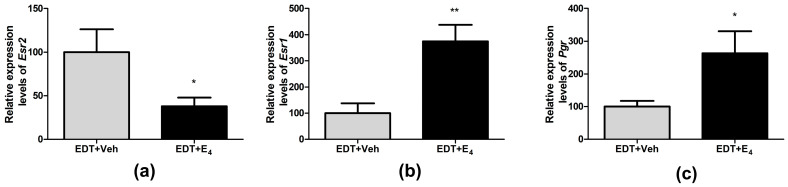
Effect of E_4_ on expression of *Esr2*, *Esr1,* and *Pgr*. Relative mRNA levels of *Esr2* (**a**), *Esr1* (**b**), and *Pgr* (**c**) were quantified by RT-qPCR in endometriotic-like lesions from endometriosis-induced mice treated for 4 weeks with E_4_ (EDT+E_4_) or vehicle-only (EDT+Veh). Statistical significance was determined by Student’s *t*-test. All data are presented as mean ± SEM (*n* = 6–8 animals per group). * = *p* < 0.05, and ** = *p* < 0.01. EDT: endometriosis; E_4_: estetrol; Veh: vehicle; *Esr2*: estrogen receptor beta gene; *Esr1*: estrogen receptor alpha gene; and *Pgr*: progesterone receptor gene.

Endometriosis is an estrogen-dependent disease, usually associated with altered expression of ERs and PR. It has been described that high levels of ERβ suppress ERα expression and contribute to secondary PR deficiency and progesterone resistance [5,6,7]. In our study, we examined hormone receptor expression, and the results revealed that the treated group with E_4_ reduced the mRNA expression of *Esr2* compared to the control group (*p* < 0.05; Figure 6a). In contrast, the mRNA expressions of *Esr1* and *Pgr* were significantly higher than in the control group (*p* < 0.01 and *p* < 0.05, respectively; Figure 6b,c). Therefore, the results suggest that treatment with E_4_ led to a correction of hormone receptor levels by decreasing the expression of *Esr2* and increasing the expression of *Esr1* and *Pgr* levels. 

## 4. Discussion

To our knowledge, this is the first study to investigate the potential therapeutic effects of E_4_ on different aspects of endometriotic-like lesion development using an in vivo model of induced endometriosis. Macroscopically, we observed that E_4_ treatment reduced the volume and weight of endometriotic-like lesions, while at the microscopic level, E_4_ increased the number of apoptotic cells without altering cell proliferation. In addition, it modulated oxidative stress and increased lipid peroxidation in favor of lesion regression. Moreover, E_4_ decreased TNF-α levels. These corrective effects of E_4_ may be related to a decrease in *Esr2* expression and an improvement in the *Esr1* and *Pgr* expression in the endometriotic tissue.

E_4_ has estrogenic activity in the uterus [28,29,30,44]. Abot et al. [30] analyzed the effect of acute E_4_ treatment at various concentrations for 24 h in ovariectomized mice and demonstrated that the steroid acts as a less potent estrogen on uterine epithelial proliferation. In addition, these authors observed that the histological changes induced by combined treatment with E_2_ (8 μg/kg) and E_4_ (200 μg/kg or 1 mg/kg) were not different from those induced by E_2_ alone (8 μg/kg). After 6 h of E_4_ treatment in ovariectomized mice, its effect on the expression of a group of uterine genes that are regulated by E_2_ in this tissue was analyzed. The study revealed that E_4_ may trigger the transcriptional activity of ERα, although doses of E_4_ approximately 100 times higher than E_2_ were necessary for the responses considered [27]. On the other hand, Visser et al. [45] found in intact rats (not ovariectomized) that E_4_ treatment for 8 weeks at a dose range of 0.5 to 3 mg/kg did not alter uterine weight. Given that we also used intact animals, at a dose of 3 mg/kg/day, and considering that the agonist potency of E_4_ is approximately 2% of the magnitude observed for E_2_ [14], this would explain why cell proliferation was not affected in the endometriotic-like lesions of animals treated with E_4_. This finding is promising, especially considering that the dose of E_4_ administered would not aggravate the condition in our experimental model. In agreement with our result, one study compared the effects of E_4_ and E_2_ in human endometriosis cell lines and primary cultures and demonstrated that incubation with E_2_ increased proliferation and migration, while E_4_ did not modify them [18]. Recently, another study has also reported that E_4_ inhibited E_2_-induced invasion and migration in immortalized human endometrial stromal cells (HESCs) [46]. Taken together, these findings are important in supporting E_4_ as a treatment for endometriosis, since cell growth, migration, and invasiveness are estrogenic effects associated with the development and progression of endometriosis. 

The lower sensitivity of endometriotic cells to apoptosis plays an important role in the development of endometriosis. Increased estrogen availability due to local E_2_ production as well as increased estrogen sensitivity due to ERβ leads to inhibition of apoptosis in endometriotic lesions [47]. In the current study, E_4_ treatment promoted apoptosis of the endometriotic cells and consequently reduced the volume and weight of endometriotic-like lesions. Even E_4_ decreased the activity of SOD and increased the activity of CAT and GPX. In endometriosis, an increase in SOD activity and a decrease in CAT activity have been reported, which leads to increased production of reactive oxygen species (ROS), promoting the proliferative phenotype [42]. Therefore, our findings suggest a decrease in the levels of H_2_O_2_, the key ROS involved in the control of cell proliferation [42,48]. They also suggest a greater accumulation of superoxide anion radical (O_2_^•−^) due to the reduction in SOD activity, favoring oxidative damage [49]. In this sense, we observed an increase in lipid peroxidation, which can potentially lead to the loss of cell membrane integrity. Thus, the above not only supports the findings of PCNA immunohistochemistry but also of the TUNEL assay and the reduced volume of lesions in treated animals. These results agree in part with the work of other authors who demonstrated the specific inhibition of SOD1 selectively (which provides 80% of the total SOD activity) promotes apoptosis of cancer cells and cell cycle arrest by elevation of the superoxide radical and reduction in hydrogen peroxide levels [50].

On the other hand, TNF-α is one of the most prominent cytokines that participate in endometriosis pathogenesis. There is evidence that indicates an aberrant function of the TNF system in endometriosis. In women without endometriosis, endometrial cells do not implant in ectopic locations because normal apoptotic mechanisms are activated by TNF-α through the TNFR1 receptor [43]. In addition, we have reported that TNFR1 deficiency promotes the growth of endometriotic-type lesions due to a low rate of apoptosis and high levels of E_2_ in mice with endometriosis induced [19]. E_2_ can promote the growth of endometriotic lesions by increasing the TNF-α levels [51]. In fact, the TNF-α levels are significantly elevated in the peritoneal fluid of patients with endometriosis [52]. Our current results show both the cytokine mRNA expression in endometriotic-like tissue and its protein levels in peritoneal fluid were significantly decreased in response to E_4_.

Estrogen receptors (ERα and ERβ) and progesterone receptors (PRs) are the key steroid receptors involved in the pathophysiology of endometriosis. A decrease in the expression of Erα and an increase in Erβ have been observed in endometriotic cells. E_2_ through ERβ overexpression promotes inflammation and inhibits the activation of apoptosis complex I, complex II, and apoptosome formation to effectively prevent TNF-α-induced apoptosis, which leads to survival of ectopic lesions [3,5]. ERβ has a key role in the development of progesterone resistance by suppressing ERα expression and contributing to secondary PR deficiency in endometriotic lesions [7]. Therefore, a reduction in ERβ levels or an increase in ERα levels could be beneficial for endometriosis treatment [18]. In fact, E_4_ treatment not only decreased *Esr2* expression but also increased *Esr1* and *Pgr* expression in the endometriotic-like lesion. Consistent with our findings, an in vitro study demonstrated incubation of endometriotic cells with E_4_ also reduced the ERβ levels and improved the ERα and PR levels, in addition to enhancing the progesterone gene response [18]. Progesterone signaling pathway limits E_2_ synthesis and inflammation. Additionally, progesterone is also able to promote cell death in endometrial and endometriotic cells [47,53]. Therefore, the increased sensitivity to progesterone of endometriotic tissue in response to E_4_ treatment could also explain the increase in apoptosis and the decrease in TNF-α.

There is strong evidence to support the use of E_4_ for clinical applications, such as contraception and menopause. E_4_ is highly bioavailable orally, with a long half-life of approximately 28–32 h, and it is not metabolized into other active estrogens [28,54,55]. Furthermore, in contrast to other estrogens, E_4_ does not bind to sex hormone binding globulin (SHBG) or increase its production by liver cells, ensuring access of the hormone to its target tissues [56]. E_4_ would have a lower thrombotic risk due to its low estrogenic effect on the liver and the balance of hemostasis, in addition to the potential to prevent bone loss [57,58]. Furthermore, some preclinical studies suggest that E_4_ has a neuroprotective effect as well as beneficial effects on cardiovascular functions [28,59]. At the therapeutic dose for hormonal therapy, E_4_ does not affect endocrine-sensitive breast cancers [60]. Additionally, E_4_ has been shown to prevent and suppress mammary tumors [45,60,61,62]. Given its proapoptotic effect in endocrine-resistant breast cancer, E_4_ might be beneficial as treatment of this pathology in postmenopausal women [61,63]. These characteristics indicate that E_4_ could be a safe steroid for use in hormone therapy.

Considering the effects of contraceptives in the treatment of endometriosis, it is worth noting that they help to reduce the large amounts of E_2_ released into the pelvic implants during ovulation [8,64]. In addition, considering that E_4_ has been shown to have a suppressive effect on ovarian activity [65] and a weak estrogenic effect in the uterus [30], it can be concluded that E_4_ could be beneficial in the treatment of endometriosis.

## 5. Conclusions

We have demonstrated that E_4_ limits the development and progression of endometriosis in vivo and reverses the alteration in hormone receptors. Together with previous studies, these findings indicate that E_4_ could be an effective and secure estrogenic compound for use as hormone therapy for endometriosis. 

To the best of our knowledge, two clinical trials are currently being developed. A Phase III clinical trial evaluates the combination E_4_/drospirenone, a combined oral contraceptive, for the treatment of endometriosis in Japanese patients with encouraging results for the relief of pelvic pain (Study No. FSN-013P-04). Another study evaluates the efficacy of E_4_/drospirenone in reducing ovarian endometriomas after 3 and 6 months of treatment (Study No. NCT05837624, date completion estimated in 2025). It is expected that the findings showed in the present preclinical study may serve to support the aforementioned studies as well as those to be developed in the future.

## Figures and Tables

**Table 1:** Primer gene symbols, sequences, GenBank access numbers, and sizes of amplicons.

Gene	GenBank Access Number	Primer Sequences (5′–3′)	Amplicon (bp)
*Esr1*	NM_001302532.1	F: CTTGGAAGGCCGAAATGAAATG R: GGCAGGGCTATTCTTCTTAGTG	103
*Esr2*	NM_207707.1	F: CTGGGTATCATTACGGTGTCTG R: GATTCGTGGCTGGACAGATATAG	99
*Pgr*	NM_008829.2	F: CCTGACACTTCCAGCTCTTT R: CGGAAACCTGGCAGAGATTTA	100
*Tnf*	NM_001278601.1	F: CTACCTTGTTGCCTCCTCTTT R: GAGCAGAGGTTCAGTGATGTAG	116
*Rn18s*	NR_003278.3	F: CTGAGAAACGGCTACCACATC R: GCCTCGAAAGAGTCCTGTATTG	107

*Esr1:* Estrogen receptor alpha gene; *Esr2*: estrogen receptor beta gene; *Pgr*: progesterone receptor gene; *Tnf*: tumor necrosis factor transcript variant 2; *Rn18s*: 18S ribosomal RNA; F: forward; and R: reverse.

## Data Availability

The raw data supporting the conclusions of this article will be made available by the authors on request.
